# Highly heterogeneous-related genes of triple-negative breast cancer: potential diagnostic and prognostic biomarkers

**DOI:** 10.1186/s12885-021-08318-1

**Published:** 2021-05-31

**Authors:** Yiduo Liu, Linxin Teng, Shiyi Fu, Guiyang Wang, Zhengjun Li, Chao Ding, Haodi Wang, Lei Bi

**Affiliations:** 1 School of Integrated Chinese and Western Medicine, Nanjing University of Chinese Medicine, 138 Xianlin Road, Nanjing, 210023 Jiangsu China; 2 College of Health Economics Management, Nanjing University of Chinese Medicine, 138 Xianlin Road, Nanjing, 210023 Jiangsu China

**Keywords:** Triple-negative breast cancer, Biomarkers, Targeted therapies, Heterogeneous-related genes

## Abstract

**Background:**

Triple-negative breast cancer (TNBC) is a highly heterogeneous subtype of breast cancer, showing aggressive clinical behaviors and poor outcomes. It urgently needs new therapeutic strategies to improve the prognosis of TNBC. Bioinformatics analyses have been widely used to identify potential biomarkers for facilitating TNBC diagnosis and management.

**Methods:**

We identified potential biomarkers and analyzed their diagnostic and prognostic values using bioinformatics approaches. Including differential expression gene (DEG) analysis, Receiver Operating Characteristic (ROC) curve analysis, functional enrichment analysis, Protein-Protein Interaction (PPI) network construction, survival analysis, multivariate Cox regression analysis, and Non-negative Matrix Factorization (NMF).

**Results:**

A total of 105 DEGs were identified between TNBC and other breast cancer subtypes, which were regarded as heterogeneous-related genes. Subsequently, the KEGG enrichment analysis showed that these genes were significantly enriched in ‘cell cycle’ and ‘oocyte meiosis’ related pathways. Four (FAM83B, KITLG, CFD and RBM24) of 105 genes were identified as prognostic signatures in the disease-free interval (DFI) of TNBC patients, as for progression-free interval (PFI), five genes (FAM83B, EXO1, S100B, TYMS and CFD) were obtained. Time-dependent ROC analysis indicated that the multivariate Cox regression models, which were constructed based on these genes, had great predictive performances. Finally, the survival analysis of TNBC subtypes (mesenchymal stem-like [MSL] and mesenchymal [MES]) suggested that FAM83B significantly affected the prognosis of patients.

**Conclusions:**

The multivariate Cox regression models constructed from four heterogeneous-related genes (FAM83B, KITLG, RBM24 and S100B) showed great prediction performance for TNBC patients’ prognostic. Moreover, FAM83B was an important prognostic feature in several TNBC subtypes (MSL and MES). Our findings provided new biomarkers to facilitate the targeted therapies of TNBC and TNBC subtypes.

**Supplementary Information:**

The online version contains supplementary material available at 10.1186/s12885-021-08318-1.

## Background

Judging from the immunohistochemistry (IHC) expression of estrogen receptor (ER), progesterone receptor (PR), and human epidermal growth factor receptor2 (HER2), breast cancer can be broadly classified into luminal-like (luminal A/B-like), HER2-positive, and triple-negative [1–4]. Triple-negative (ER-, PR-, and HER2-) breast cancer (TNBC), a heterogeneous disease, comprises approximately 10–20% of all breast cancers [5–8] and can be further grouped into distinct subtypes by gene expression profiling [9]. TNBC has higher histopathological grade, proliferation index and risk of invasion than other breast cancer subtypes [10, 11], posing a great challenge for public health.

Due to the peculiar phenotype of TNBC, patients with the conventional anti-cancer treatment strategies may not show effective outcomes. Chemotherapy currently is the standard treatment [12–14], while TNBC patients frequently develop resistance to the chemotherapy drugs [15–17]. Over 50% are likely to recur after chemotherapy regimens [18, 19], which gives rise to great difficulty for the treatment of TNBC. Furthermore, the accurate diagnosis of early-stage TNBC is still tricky, and the advanced stage may accompany a poor prognosis [7, 10, 20]. Previous studies reported that the 5-year survival rate of TNBC patients dramatically decreased from stage II to stage III (from 76% to 45%) [21], which suggested that early diagnosis could improve the survival rate of TNBC [22]. Hence, it is urgent to find the specific and sensitive biomarkers to diagnose TNBC.

**Fig. 1:**
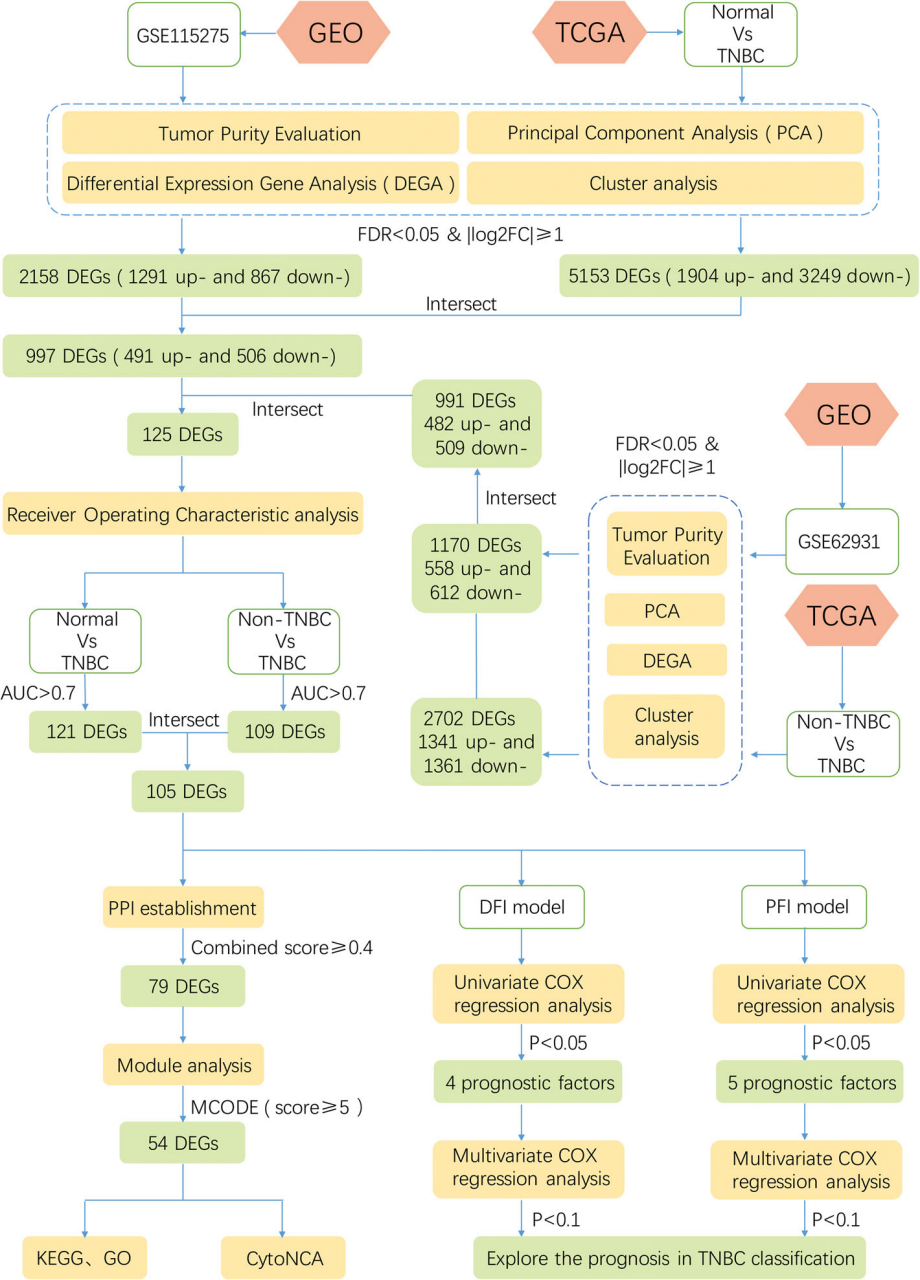
The workflow for identification of potential diagnostic and prognostic biomarkers in triple-negative breast cancer

Recently, bioinformatics methods were widely used in cancer research and applied to study the molecular mechanisms of diseases [23–28]. The pathogenesis of TNBC may be a series of modifications of genetic, genomic and expression profiles [29]. Therefore, transcriptome based genetic perspective research is helpful to understand the occurrence and development of TNBC [30]. Biomarkers may have a great accurate prediction of metastatic behaviors in TNBC [31]. Furthermore, they can evaluate the diagnosis and prognosis of TNBC subtypes [32]. Overall, bioinformatics would contribute to the precise treatment of TNBC. In this study, transcriptome data were used to explore potential biomarkers of TNBC and TNBC subtypes. The workflow showed in Fig. 1.

## Methods

### Acquisition of gene expression data and patient's clinical information

Gene expression microarray datasets were obtained from the GEO database (https://www.ncbi.nlm.nih.gov/geo/) according to the following requirements: (1) dataset was mRNA expression profile data; (2) the information of the platform annotation was accessible; (3) the expression data of microarray had been published. Therefore, the datasets including GSE115275 [33] and GSE62931 [34] were downloaded by GEO query package in R software (version 3.6.1, https://www.r-project.org). Meanwhile, mRNA sequencing data of 1247 samples of breast cancer and normal breast tissue were acquired from the TCGA database (http://portal.gdc.cancer.gov/). Subsequently, the data from TCGA and GEO datasets were filtered, and a total of 120 normal tissue samples, 176 TNBC samples, and 916 non-TNBC samples were finally brought into our study (Additional file 1: Table S1).

### Differential expression gene analysis

Before differential expression gene (DEG) analysis, tumor purity estimation and principal component analysis (PCA) were used to evaluate the samples. The limma package was used to perform DEG analysis, and DEGs (FDR < 0.05 & |log2FC| ≥ 1) were considered to be significantly changed. Then, the up- and down- regulated DEGs were further verified in sequencing data of TCGA.

### Receiver operating characteristic curve analysis and enrichment analysis

The plotROC package was employed to predict the ability of DEGs to identify TNBC from breast cancer via Receiver Operating Characteristic (ROC) curve analysis. DEGs with Area Under the Curve (AUC) higher than 0.7 were further used for GO and KEGG (www.kegg.jp/kegg/kegg1.html) [35, 36] pathway enrichment analyses through FunRich (version 3.1.3, http://www.funrich.org) software [37].

### Protein-protein interaction network and module analysis

In the meanwhile, DEGs with discriminative ability to TNBC were used to download protein interaction information from STRING (https://string-db.org/). After that, Cytoscape (version 3.6.0, https://cytoscape.org) software was employed to build the protein-protein interaction (PPI) network. Molecular Complex Detection (MCODE) plug-in was used to detect modules in the network [38, 39]. Models with score ≥ 5 were considered for further enrichment analysis, which could help to explore the potential biological functions of DEGs. Subsequently, the CytoNCA was applied to evaluate betweenness centrality (BC), degree centrality (DC), and closeness centrality (CC) of the nodes in the network modules [40].

### Univariate and multivariate cox regression analysis

Survival package in R software was performed to univariate Cox regression analysis of DEGs, and DEGs with *p* < 0.05 were considered as prognosis signatures. Then, multivariate Cox regression models for the DFI (DFI defined as the time between the treatment of primary cancer and the development of regional metastases [41]) and PFI (PFI defined as the time from randomization to the first documentation of objective tumor progression or death [42]) were established based on these DEGs. And the risk score was calculated according to the following formula: risk score = ${\sum}_{i=1}^n\left({coef}_i\ast {Expr}_i\right)$, where *coef* was the regression coefficient of genes and *expr* represented the gene expression values. According to the median risk score, TNBC samples were divided into high- and low-risk groups. Besides, the time-dependent ROC curve was plotted, which presented the predictive accuracy of multivariate Cox regression models. Furthermore, rms package was used to integrate the clinical information (including age, pathological stage, menopause and ethnicity) into the multivariate Cox regression model, and the sensitivity and specificity of the model were calculated.

### Survival analyses of TNBC subtypes

TNBCtype (https://cbc.app.vumc.org/tnbc/) tool [43] and the TNBC subtype-specific genes [44] were respectively used to identify TNBC subtypes. Six distinct subtypes were discovered by TNBCtype, including two basal-like (BL1 and BL2), an immunomodulatory (IM), a mesenchymal (M), a mesenchymal stem-like (MSL), and a luminal androgen receptor (LAR). And four TNBC clusters, such as Luminal Androgen Receptor (LAR), mesenchymal (MES), basal-like immunosuppressed (BLIS), and basal-like immune-activated (BLIA) were obtained based on TNBC subtype-specific genes. Finally, the prognostic value of TNBC prognostic factors was evaluated according to these classifications.

## Results

### Identification of DEGs in TNBC tissue samples

**Fig. 2:**
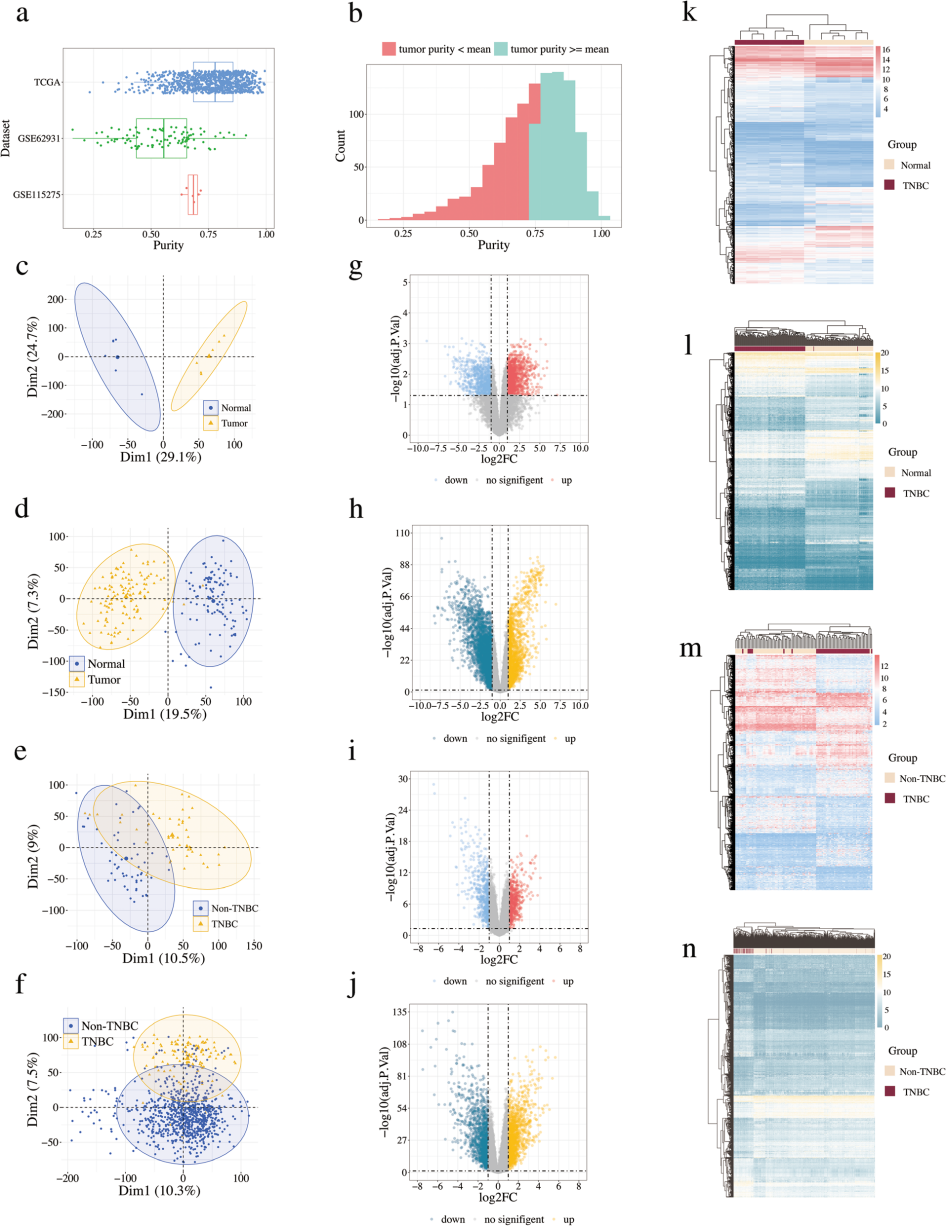
The results of DEGs analyses in GSE62931, GSE115275 and TCGA cohort. **a** Boxplot of tumor purity in TCGA, GES115275 and GES62931. **b** Histograms of tissues’ tumor purity. PCA plot of **c** GSE115275, **d** normal and TNBC samples from TCGA, **e** GSE62931, **f** non-TNBC and TNBC samples from TCGA. Volcano plot of **g** GSE115275, **h** normal and TNBC samples from TCGA, (**i**) GSE62931, (**j**) non-TNBC and TNBC samples from TCGA. Upregulated genes and downregulated genes are represented by red and blue dots in the volcano map, however, the genes having no obvious difference are represented by gray. Heatmap of (**k**) GSE115275, (**l**) normal and TNBC samples from TCGA, (**m**) GSE62931, (**n**) non-TNBC and TNBC samples from TCGA

The tumor purity of 1092 breast cancer tissues were between 0.159 and 0.994, and 56.4% of them had a purity value greater than the mean of 0.741 (Fig. 2a-b). Additionally, the results of PCA demonstrated that in the selected datasets, the experimental group and the control group could be well distinguished (Fig. 2c-f), which suggested the selected samples were suitable for further analysis. As shown in Fig. 2g-j, 2158 DEGs were identified between TNBC and normal breast tissues (FDR < 0.05 and |log2FC| ≥ 1) were identified in GSE115275, including 1291 up-regulated genes and 867 down-regulated genes, while 1170 DEGs between TNBC and non-TNBC (558 up-regulated genes and 612 down-regulated genes) were obtained from GSE62931. After validation with the TCGA database, 991 DEGs (482 up-regulated genes and 509 down-regulated genes) between TNBC and non-TNBC, and 997 DEGs (491 up-regulated genes and 506 down-regulated genes) between TNBC and non-TNBCnormal breast tissues were screened out. The results were shown in Fig. 2k-n, which illustrated that these DEGs could be used to cluster different groups of samples. Finally, 125 common genes were obtained by intersecting the DEGs between TNBC and normal breast tissues and DEGs between TNBC and non-TNBC (Additional file 2: Table S2).

### ROC analysis of DEGs

**Fig. 3:**
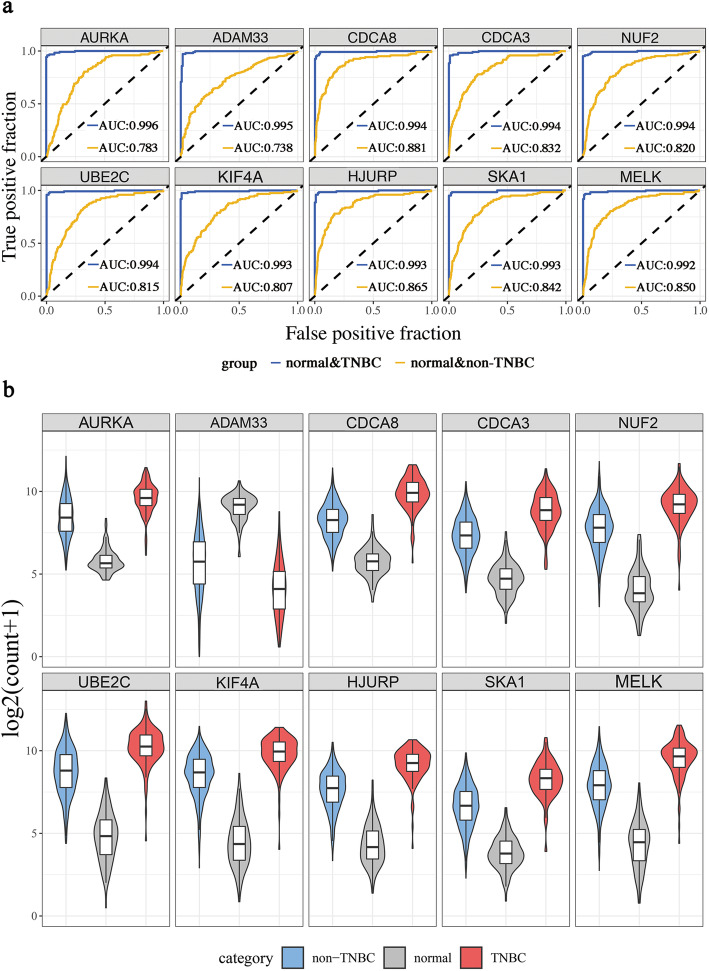
The top ten heterogeneous-related genes screened out from DEGs

For further screening, the verification efficiency of the 125 DEGs was measured using the ROC curve analysis. Among them, 121 DEGs had the good talent to distinguish normal breast tissues and TNBC samples, while 109 DEGs had superior performance in distinguishing between non-TNBC samples and TNBC samples. The intersection of these DEGs was taken to get 105 heterogeneous-related genes that could specifically identify TNBC (Additional file 3: Table S3). As shown in Fig. 3a, based on the AUC values, the top 5 genes were AURKA, ADAM33, CDCA8, CDCA3 and NUF2. Moreover, the expression of these genes in non-TNBC and TNBC was higher than that in normal tissues (Fig. 3b).

### Enrichment analysis of heterogeneous-related genes

**Fig. 4:**
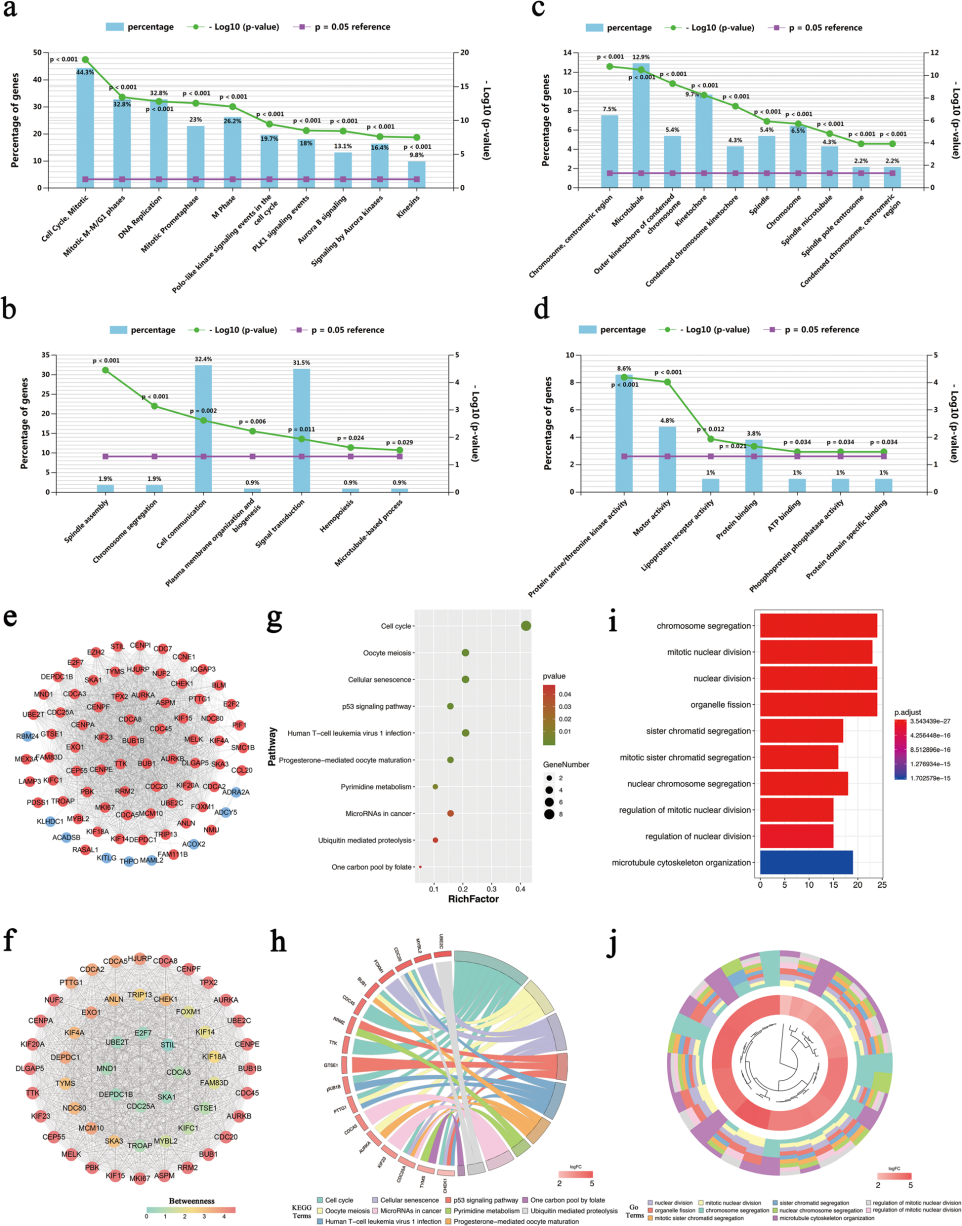
GO and KEGG analysis of heterogeneous-related genes. **a** Biological pathway of heterogeneous-related genes. **b** Biological process of heterogeneous-related genes. **c** Cellular component of heterogeneous-related genes. **d** Molecular function of heterogeneous-related genes. **e** PPI network of heterogeneous-related genes. Upregulated genes are represented by red nodes and downregulated genes are represented by blue nodes. **f** Module, MCODE score ≥ 5 1 of PPI network. **g**-**h** Pathway analysis of module 1. **i**-**j** Functional enrichment analysis of module 1

We then performed GO functional annotation and KEGG pathway enrichment analysis of the heterogeneous-related genes (Additional file 4: Table S4) were performed then. As shown in Fig. 4a, KEGG pathway enrichment analysis revealed that they were primarily enriched in ‘cell cycle, mitotic’, ‘mitotic M-M/G1 phases’, and ‘DNA replication’ related pathways. In terms of biological process, these genes were significantly enriched in ‘spindle assembly’, ‘chromosome segregation’ and ‘cell communication’ (Fig. 4b). As for cellular composition, the heterogeneous-related genes were significantly related to ‘chromosome, centromeric region’, ‘microtubule’, and ‘outer kinetochore of condensed chromosome’ (Fig. 4c). Additionally, these genes were mainly involved in ‘Pprotein serine/threonine kinase activity’, ‘motor activity’ and ‘lipoprotein receptor activity’ of molecular functions (Fig. 4d).

### PPI network establishment and pathway analysis of network module

Through inputting the 105 heterogeneous-related genes into the STRING, the PPI network of 79 genes was constructed (combined score > 0.4, Fig. 4e). The highest-scoring module (module 1), a cluster with score = 50.755, was detected by MCODE, which includingincluded 54 genes. Furthermore, the hub nodes (DLGAP5, KIF20A, CENPA, NUF2, and CDCA8) of module 1 were determined by CytoNCA (Fig. 4f). Finally, according to enrichment analysis, the heterogeneous-related genes in module 1 were enriched in ‘cell cycle’ and ‘oocyte meiosis’ related pathways, as well as ‘chromosome segregation’ and ‘mitotic nuclear division’ related gene functions (Fig. 4g-j).

### Univariate cox regression analysis and identification of prognostic factors

**Fig. 5:**
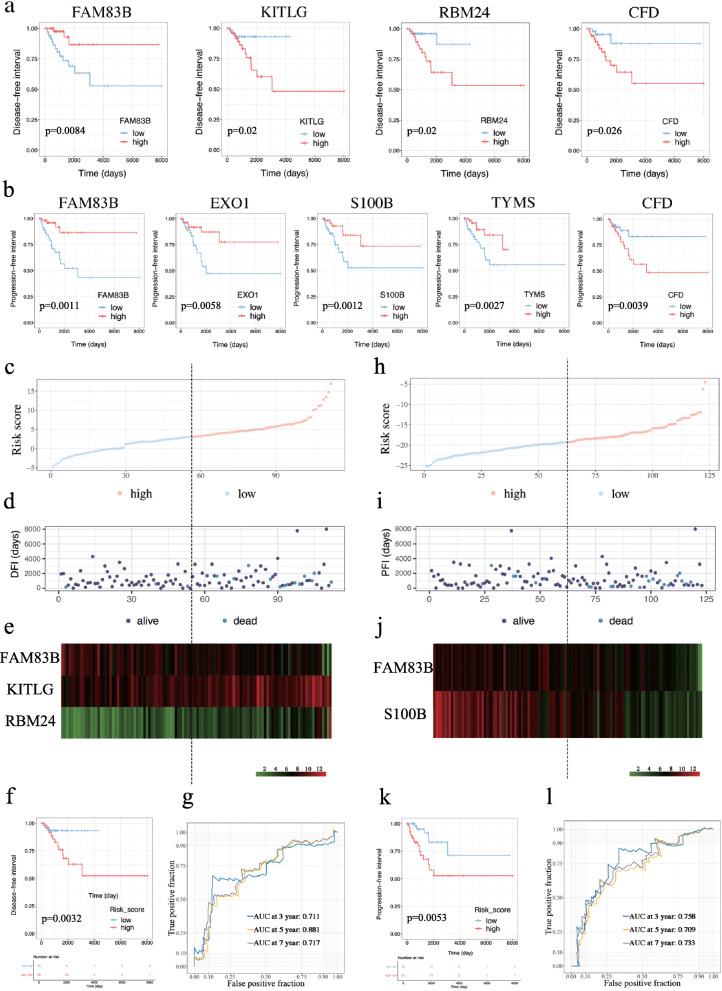
The result of Cox regression analysis of TNBC patients in the DFI and PFI models. **a** Kaplan-Meier curves of analysis of DFI for FAM83B, KITLG, RBM24 and CFD. **b** Kaplan-Meier curves of PFI for FAM83B, EXO1, S100B, TYMS and CFD. **c** Volcano plot of risk score of high and low risk TNBC patients in the DFI model. **d** Scatter plot of survival status and time of TNBC patients in the DFI model. **e** Expression of FAM83B, KITLG and RBM24 in high-risk and low-risk groups in the DFI model. **f** Kaplan-Meier curve for DFI in low-risk and high-risk groups. **g** 3-year, 5-year and 7-year time-dependent ROC curves of the DFI model. **h** Volcano plot of risk score of TNBC patients in the PFI model. **i** Scatter plot of survival status and time of TNBC patients in the PFI model. **j** Expression of FAM83B and S100B in high-risk and low-risk groups in the PFI model. **k** Kaplan-Meier curve for PFI in low-risk and high-risk groups. **l** Time-dependent ROC for 3-year, 5-year and 7-year PFI in TNBC patients 3-year, 5-year and 7-year time-dependent ROC curves of the PFI model

To identify prognostic features among the 105 heterogeneous-related genes in TNBC patients’ DFI and PFI, the univariate Cox regression models and plotted Kaplan-Meier curves were constructed accordingly. As shown in Fig. 5a-b and Additional file 5: Table S5, four genes (FAM83B, KITLG, CFD and RBM24) were significantly (*p* < 0.05) correlated with the DFI of 109 TNBC patients and five genes (FAM83B, EXO1, S100B, TYMS and CFD) significantly (*p* < 0.05) affected the PFI of 120 TNBC patients. As shown in Additional file 6: Table S6, among four critical clinical parameters (age, menopause, ethnicity, and the pathological stage), and found that the pathological stage was a prognostic factor in both DFI and PFI models (*p* < 0.05).

### Multivariate cox regression analysis and establishment of prognostic risk model

Based on the results from the above analysis, multivariate Cox regression analysis was performed to establish a DFI model with the four prognostic features. The following formula was used to calculate the risk score: risk score = expression of FAM83B ∗ (− 1.584) + expression of KITLG ∗ 1.225 + expression of RBM24 ∗ 1.182. After ranking the risk scores, the result showed that the high-risk group showed high expression of KITLG and RBM24 as well as low expression of FAM83B (Fig. 5c-e). As shown in Fig. 5f, survival analysis manifested that the high-risk groups exhibited worse prognosis than the low-risk groups. Furthermore, the corresponding AUC value of the risk score for the 3-year, 5-year, and 7-year DFI were 0.711, 0.702, and 0.717, respectively (Fig. 5g).

Besides, we also performed multivariate Cox regression analysis was performed according to the prognostic factors of PFI. The calculation formula was as follows: risk score = expression of FAM83B ∗ (− 1.437) + expression of S100B ∗ (− 1.020). As shown in Fig. 5h-k, high risk scores were associated with by poor prognosis. Meanwhile, the AUC values of 3-, 5- and 7-year (0.758, 0.709 and 0.733) were shown in Fig. 5l, which suggested that the risk score had a good predictive ability for prognosis.

### Clinic correlation and of prognostic factors

**Fig. 6:**
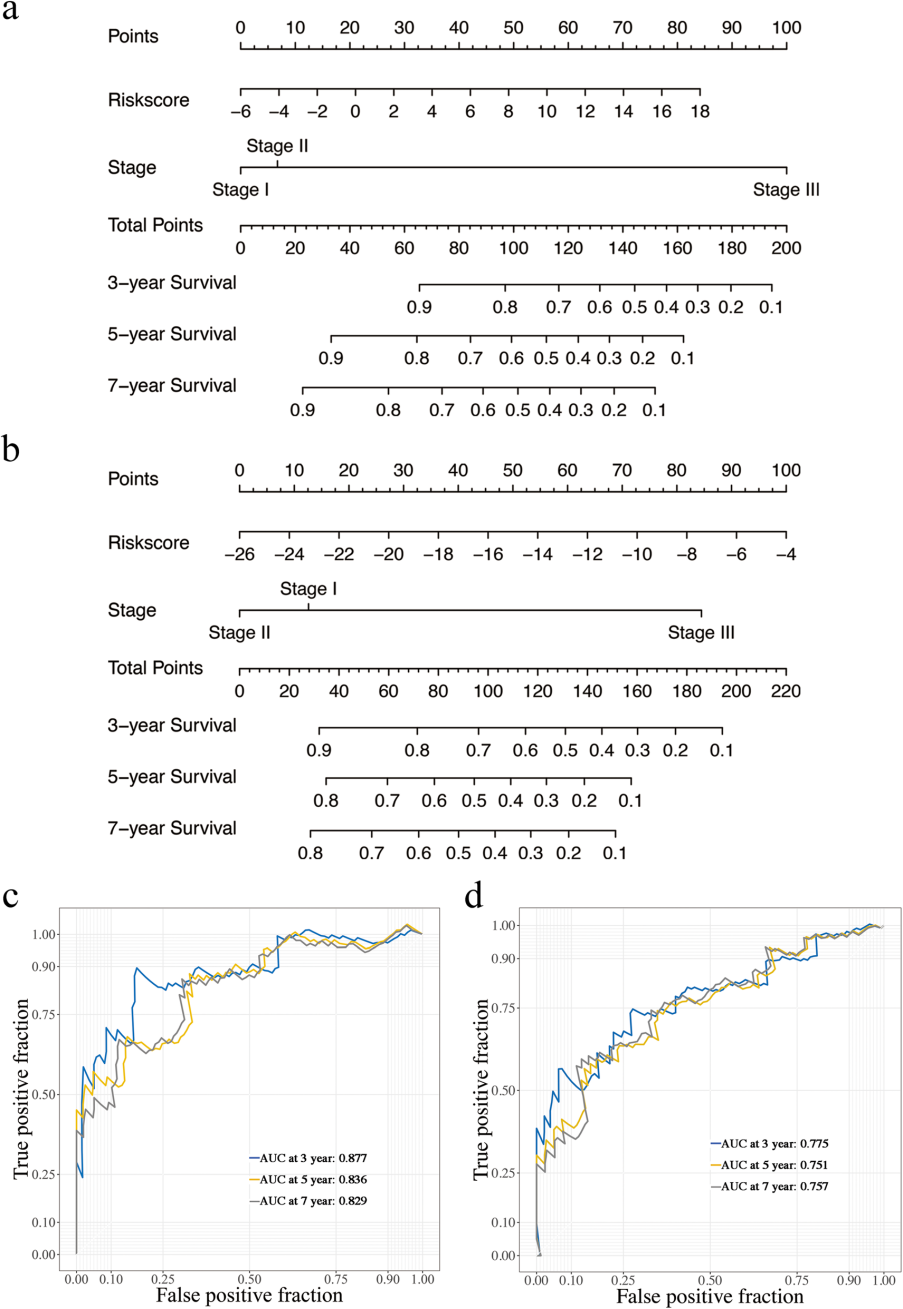
Establishment and evaluation of nomogram model. **a**-**b** The nomograms for DFI and PFI. **c**-**d** 3-year, 5-year and 7-year time-dependent ROC curves of the nomogram model

Nomograms were plotted to further evaluate the predictive significance of the clinical prognosis factor, combining the results of multivariate Cox regression analysis (Fig. 6a-b). As shown in Fig. 6c, the AUC values of the 3-, 5-year and 7-year DFI were 0.877, 0.836 and 0.829, respectively, and 0.775, 0.751 and 0.757 for PFI for 3-, 5- and 7-years, respectively (Fig. 6d). It indicated that the nomograms had a good predictive performance of TNBC patients.

### Survival analyses of TNBC subtypes

**Fig. 7:**
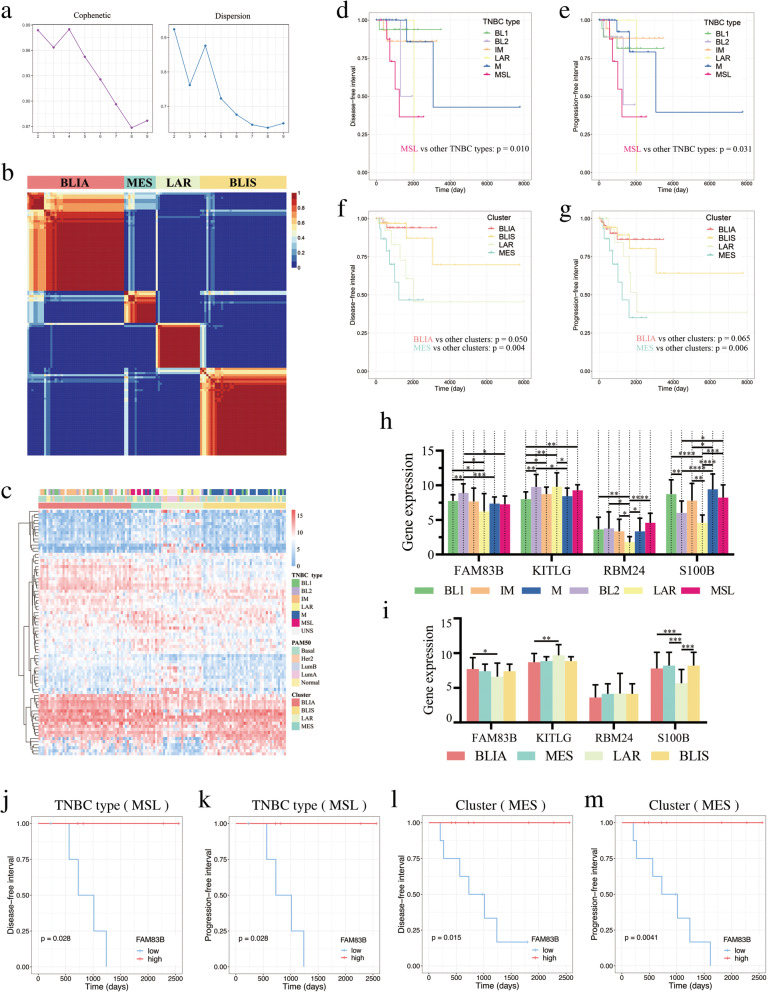
Survival analysis of TNBC subtypes. **a** Cophenetic and dispersion metrics for 2–9 clusters. **b** Consensus plots for rank basis four NMF clusters. **c** Heatmap of gene expression of four clusters. **d**-**e** Kaplan-Meier curves for DFI and PFI in different TNBC types. **f**-**g** Kaplan-Meier curves for DFI and PFI in different clusters. **h**-**i** Expression of FAM83B, KITLG, RBM24 and KITLG in different TNBC types and clusters. *p < 0.05, ** *p* < 0.01, *** *p* < 0.001 and **** *p* < 0.0001. (**j**-**m**) DFI and PFI Kaplan-Meier curves of FAM83B

Based on the Non-negative Matrix Factorization (NMF) algorithm, TNBC tissues were divided into four clusters stably (Fig. 7a-b). As shown in Fig. 7c, (1) all MSL and LAR TNBC subtypes were included in MSL and LAR clusters respectively; (2) most IM and M were included in BLIA and BLIS clusters respectively; (3) BL1 appeared in both BLIA and BLIS. The result of survival analysis showed that BLIA had a better prognosis than other TNBC subtypes, whereas MSL and MES clusters were associated with poor prognosis (Fig. 7d-g). Moreover, the expression differences of FAM83B, KITLG, RBM24 and S100B between different classifications were shown in Fig. 7h-i. Of note, compared with LAR (both in TNBC subtypes and TNBC clusters), S100B was significantly up-regulated in IM (BLIA), MSL (MES) and M (BLIS). Thus, S100B might be used for TNBC subtypes identification. Not only that, our results showed that FAM83B significantly affected the prognosis of both MSL and MSE (*p* < 0.05, Fig. 7j-m). Moreover, KITLG, RBM24 and S100B also had an effect on the prognosis of some TNBC subtypes with no significant difference (0.05 < *p* < 0.2, Additional file 7: Fig. S1).

## Discussion

TNBC displays a worse prognosis than other breast cancer subtypes on account of more aggressive behaviors, higher risk of recurrences and the absence of targeted therapeutic options [10, 11, 45, 46]. The present study, we focused on heterogeneous-related genes of TNBC to find new biomarkers, which might facilitate the development of TNBC treatments.

Our result showed that 105 heterogeneous-related genes had good performances in distinguishing TNBC from other breast cancer subtypes. The top 5 of them were AURKA, ADAM33, NUF2, CDCA3 and CDCA8. AURKA is a key regulator of mitosis and a kinase that promotes cancer migration and invasion [47]. It might facilitate TNBC cell proliferation by regulating the ‘cell cycle, mitotic’ signaling pathway (Fig. 4a) and played a major role in promoting TNBC metastatic phenotype [48]. It’s believed that ADAM33 may inhibit aggressiveness and metastasis inof TNBC, thus it has the function of tumor suppression [49, 50]. NUF2 may regulate the carcinogenesis and progression of breast cancer via ‘cell cycle’ related pathways [51]. Recent studies also showed that the expression level of NUF2 was higher in TNBC than that in non-TNBC [52]. Cell division cycle-associated (CDCA) genes could lead to malignancy through dislocating cell division [53–55]. High expression of CDCA3 and CDCA8 had been found in breast cancer, resulting in a poor prognosis [55]. Of note, compared with normal tissues, these five genes showed the same regulation trend in TNBC and non-TNBC, especially biggish in TNBC (Fig. 3b), which might lead to higher malignancy of TNBC andthan other phenotypes.

DFI and PFI have crucial roles in assessing cancer prognosis once the disease has recurred [56, 57]. Thus, DFI and PFI were considered as outcome indicators in this study. In the DFI model, up-regulated KITLG, RBM24 and CFD were correlated with poor prognoses, while FAM83B exhibited the contrary effect. Moreover, high expression of FAM83B, EXO1, S100B and TYMS prolonged the PFI, while CFD shortened the PFI of TNBC patients. A recent study suggested FAM83B could inhibit cancer chemotherapy resistance through inhibiting the Wnt pathway [58]. However, FAM83B might activate the MAPK and PI3K/Akt pathways [59, 60]. Up-regulated FAM83B could cause poor OS in pancreatic ductal adenocarcinoma (PDAC) [61] and non-small cell lung cancer (NSCLC) [62]. Overall, little is known about the role of FAM83B in cancers, especially in TNBC. Our result showed that FAM83B could be a prognostic feature of both TNBC and TNBC subtypes. Therefore, it is urgently required to explore the mechanism between FAM83B and TNBC development. There was a negative correlation between FAM83B and CFD (Additional file 8: Fig. S2), which indicated that they might play opposite roles in the progression of TNBC. Complement factor D (CFD) was a rate-limiting enzyme [63], implicating in the alternative complement pathway activation and involving in an innate immune pathway in numerous cancers [63–65]. Like CFD, KITLG could also be considered as a predictive marker for multiple cancers [66, 67]. Our findings showed CFD and KITLG (an immunostimulatory molecule [68]) enriched in the ‘immune system’ pathway (Additional file 4: Table S4). This pathway could influence breast tumor progression and immune surveillance [69]. Thus, it could be seen that they might synergistically promote breast cancer progression. S100B is an inflammatory mediator [70]. The previous study had demonstrated that a high S100B expression predicted a good distant metastases-free survival in all patients with breast cancer [70], which was in line with our result.

EXO1 and TYMS were closely correlated with CFD (Additional file 8: Fig. S2). TYMS plays a crucial role in DNA synthesis and repair [71]. And a recent study demonstrated that a high TYMS level predicted a good prognosis in TNBC patients [72]. EXO1 is a core gene of DNA metabolism [73]. Its polymorphism is related to clinical outcome and susceptibility of multiple tumors [74, 75]. Qi, L. et al. [76] suggested that a higher expression level of EXO1 came with a shorter OS in TNBC. We also confirmed the relationship between EXO1 expression and OS in TNBC (Additional file 9: Table S7), and the result was the same as that of Qi, L. et al. It has been reported that PFI might not be positive correlated with OS [77], which seems to be reasonable that the high level of EXO1 accompanied by long PFI in our result. Collectively, TYMS might be considered as a potential biomarker of TNBC, but the role of EXO1 was waiting for the further reveal.

RBM24 might relate to ‘regulation of nucleobase, nucleoside, nucleotide and nucleic acid metabolism’ of biological process (Additional file 4: Table S4). What’s more, several studies demonstrated that RBM24 might regulate the stability of mRNA transcripts in different human cancer cell lines [78, 79]. Thus, it could be speculated that RBM24 might have an effect on promoting cancer. However, the association between RBM24 and TNBC is not yet well understood. It warranted further investigation regarding its precise molecular mechanism.

In conclusion, our study provided potential biomarkers for TNBC and TNBC subtypes, which might provide new treatment strategies for the research and clinical treatment of TNBC. However, this study had several limitations as well. First of all, we were unable to determine the reasons for the significant changes of the heterogeneous-related genes in TNBC patients (i.e., we cannot distinguish between expression changes that are due to tumor-specific mutations or epigenetic modification). Secondly, because the publicly available gene microarray from different databases could inevitably introduce bias, our conclusions need to be verified in more datasets. Finally, the findings of the present study need to demonstrate in vivo and in vitro experiments.

## Conclusions

In conclusion, we identified nine (four for DFI, five for PFI) of 105 heterogeneous-related genes were identified that acted as promising diagnostic and prognostic biomarkers in patients with TNBC via integrated bioinformatics analysis. It is worth mentioning that FAM83B might be further used to predict the prognosis of MSL and MES subtypes. Both DFI and PFI models presented great performances in predicting 3-, 5-, and 7-year prognoses. Our findings may provide new diagnostic and prognostic biomarkers to facilitate the targeted therapies of TNBC and TNBC subtypes.

## Supplementary Information

**Additional file 1: Table S1.** Statistics of microarray datasets from GEO and TCGA.

**Additional file 2: Table S2.** The DEGs in GEO and TCGA datasets.

**Additional file 3: Table S3.** ROC analysis of DEGs based on TCGA samples.

**Additional file 4: Table S4.** Related information of all genes involved in GO and KEGG analysis.

**Additional file 5: Table S5.** Related information of all genes involved in univariate Cox regression analysis.

**Additional file 6: Table S6.** The clinical data and the result of Cox regression analysis in DFI and PFI models.

**Additional file 7: Figure S1.** Survival analysis of TNBC subtypes.

**Additional file 8: Figure S2.** Scatter plots of correlation analysis of prognostic factors.

**Additional file 9: Table S7.** OS analysis of TNBC and Breast cancer.

## Data Availability

The datasets generated and/or analysed during the current study are available in the Gene Expression Omnibus (GEO) https://www.ncbi.nlm.nih.gov/geo/), R software (version 3.6.1, https://www.r-project.org), The Cancer Genome Atlas (TCGA) http://portal.gdc.cancer.gov/), The Gene Ontology (GO) http://geneontology.org/), KEGG database (https://www.kegg.jp/kegg/kegg1.html), FunRich (version 3.1.3, http://www.funrich.org), STRING (https://string-db.org/), Cytoscape (version 3.6.0, https://cytoscape.org), and TNBCtype (https://cbc.app.vumc.org/tnbc/). The RNA-seq raw data are available at the GEO database under accession number GSE115275 and GSE62931.

## Abbreviations

TNBC: Triple-negative breast cancer
DEG: Differential expression gene
ROC: Receiver operating characteristic
PPI: Protein-protein interaction
DFI: Disease-free interval
PFI: Progression-free interval
IHC: immunohistochemistry
PR: Progesterone receptor
ER: Estrogen receptor
HER2: Human epidermal growth factor receptor2
GEO: Gene Expression Omnibus
TCGA: The Cancer Genome Atlas
PCA: Principal component analysis
AUC: Area under the curve
MCODE: Molecular Complex Detection
DC: Degree centrality
CC: Closeness centrality
BC: Betweenness centrality
BL1/2: Basal-like 1/2
IM: Immunomodulatory
M: Mesenchymal
MSL: Mesenchymal stem–like
LAR: Luminal androgen receptor
BLIS: Basal-like immunosuppressed
BLIA: Basal-like immune-activated
NMF: Non-negative Matrix Factorization
CDCA: Cell division cycle-associated
PDAC: Pancreatic ductal adenocarcinoma
NSCLC: Non-small cell lung cancer
CFD: Complement factor D
