# Supplementary figures and images for: Highly heterogeneous-related genes of triple-negative breast cancer: potential diagnostic and prognostic biomarkers
Source: BMC Cancer. 2021 May 31;21:644. doi: 10.1186/s12885-021-08318-1 (PMC8165798; doi:10.1186/s12885-021-08318-1)

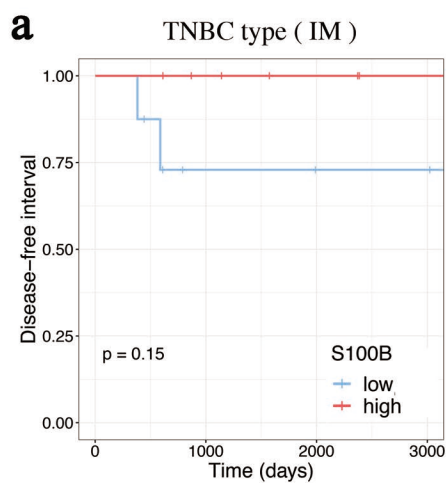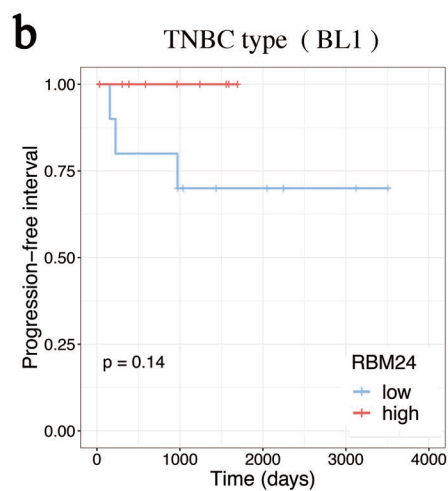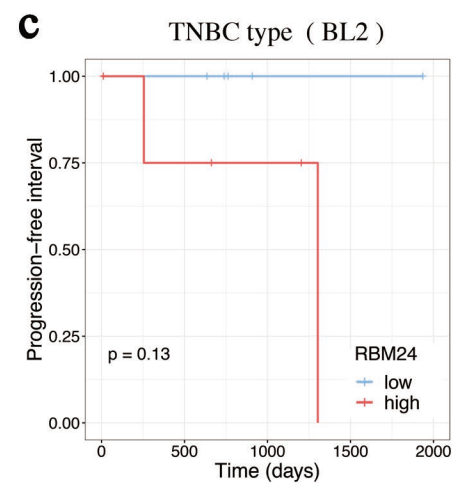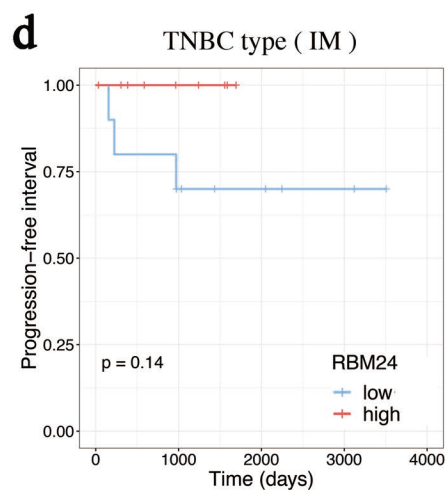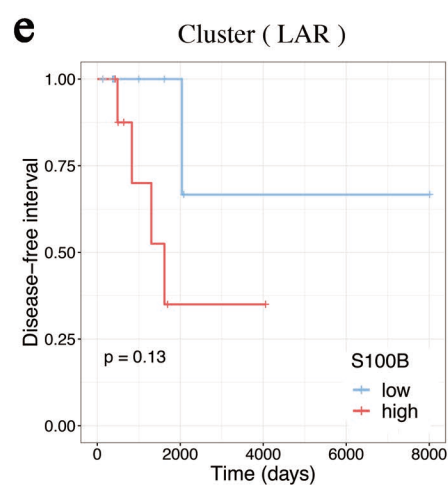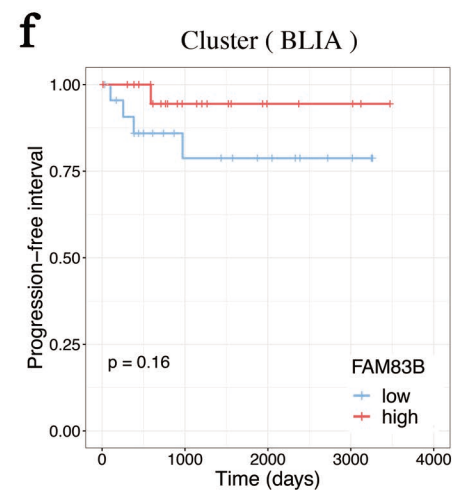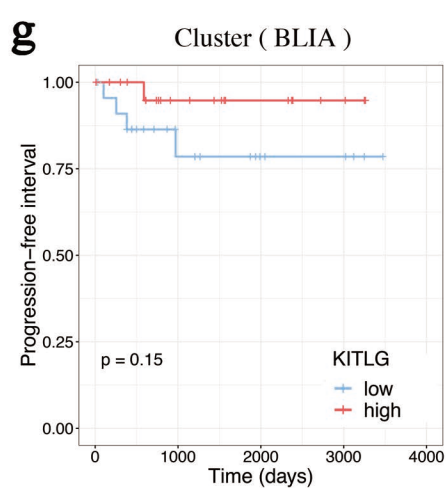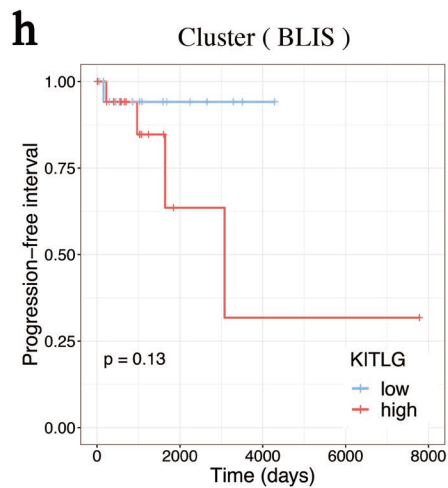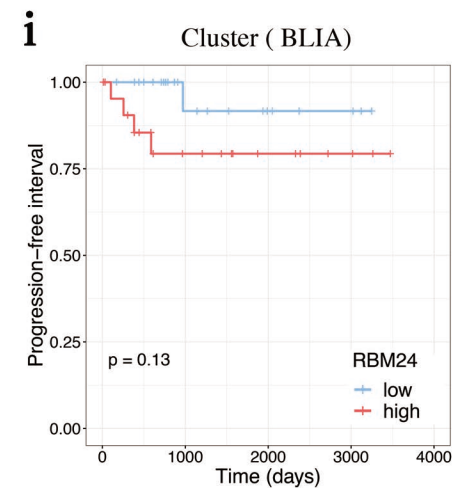

Supplement: Supplementary file 7 — Additional file 7: Figure S1. Survival analysis of TNBC subtypes. [file 12885_2021_8318_MOESM7_ESM.pdf]

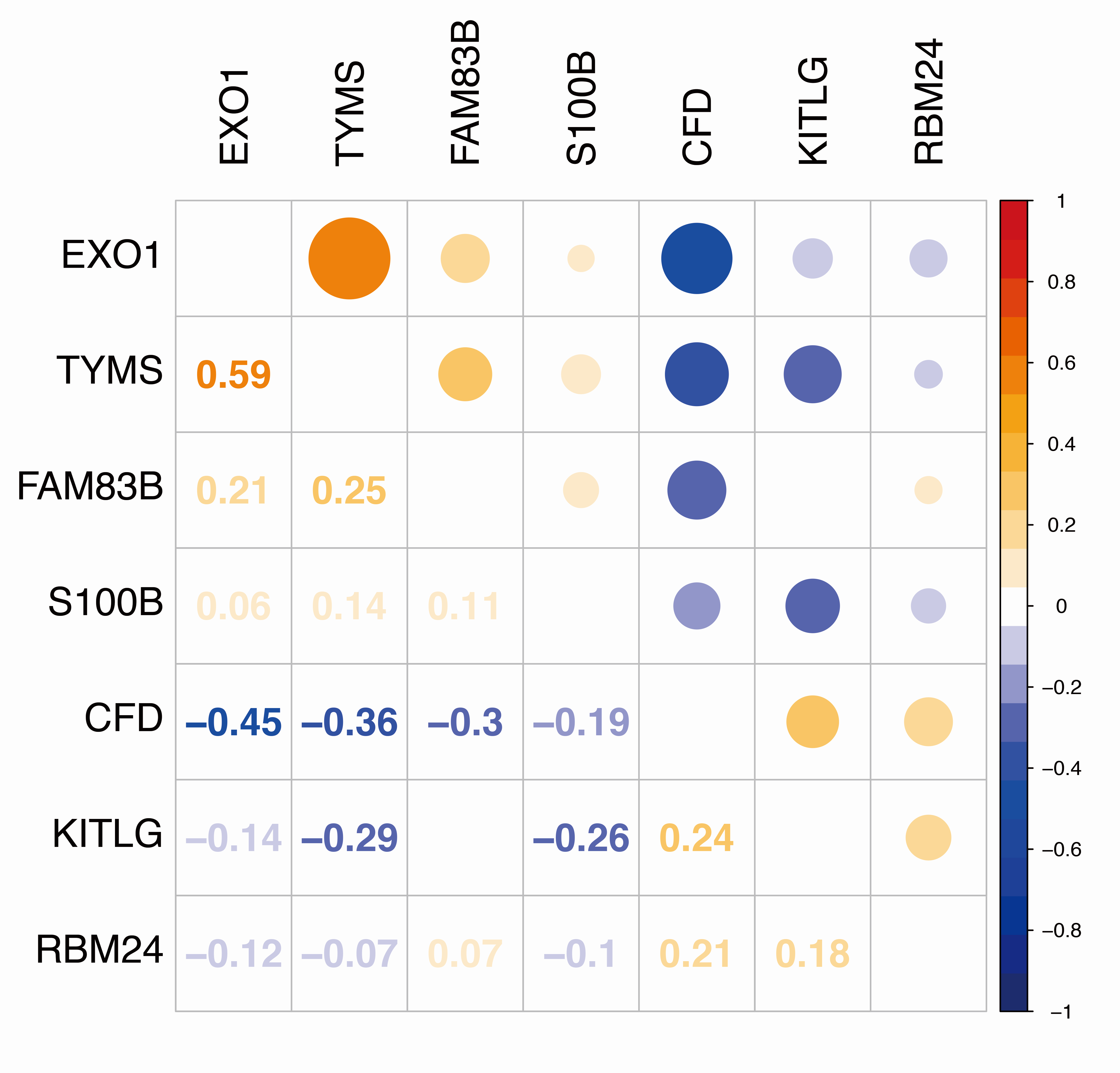

Supplement: Supplementary file 8 — Additional file 8: Figure S2. Scatter plots of correlation analysis of prognostic factors. [file 12885_2021_8318_MOESM8_ESM.tif]
